# Supplementary material for: Room temperature exciton–polariton Bose–Einstein condensation in organic single-crystal microribbon cavities
Source: Nat Commun. 2021 Jun 1;12:3265. doi: 10.1038/s41467-021-23524-y (PMC8169864; doi:10.1038/s41467-021-23524-y)
Supplement: Supplementary file 1 — Supplementary Information [file 41467_2021_23524_MOESM1_ESM.pdf]

Supplementary Information

Room temperature exciton-polariton Bose-Einstein condensation in organic single-crystal microribbon cavities

Ji Tang[1,2], Jian Zhang[3], Yuanchao Lv[1], Hong Wang[1,2], Fa Feng Xu[1,2], Chuang Zhang[1,2], Liaoxin Sun[3], Jiannian Yao[1,2], & Yong Sheng Zhao[1,2]*

[1]Key Laboratory of Photochemistry, Institute of Chemistry, Chinese Academy of Sciences, Beijing 100190, China.

[2]University of Chinese Academy of Sciences, Beijing 100049, China.

[3]State Key Laboratory of Infrared Physics, Shanghai Institute of Technical Physics, Chinese Academy of Sciences, Shanghai 200083, China.

*e-mail: yszhao@iccas.ac.cn

Supplementary Figures

Supplementary Figure 1 | Synthesis route of PDI-O.

Supplementary Figure 2 | Calculated transition dipole moment vector of PDI-O from S_0 to S_1.

Supplementary Figure 3 | Normalized absorption and PL spectra of PDI-O dilute solution.

Supplementary Figure 4 | The bright-field image of a PDI-O microribbons.

Supplementary Figure 5 | AFM image and the corresponding cross section profile of a single PDI-O microribbon.

Supplementary Figure 6 | PL spectra of a PDI-O microribbon.

Supplementary Figure 7 | XRD patterns of the PDI-O microribbons (black) and simulated PDI-O single crystals (red).

Supplementary Figure 8 | A unit cell of PDI-O crystal.

Supplementary Figure 9 | PL intensity as a function of excitation polarization angle with respect to the PDI-O microribbon long axis.

Supplementary Figure 10 | Simulated electric field intensity distribution in a microribbon with a dipole source.

Supplementary Figure 11 | AR-μ-PL spectra of microribbons with different width (W) and thickness (d).

Supplementary Figure 12 | Relevant energy levels of PDI-O molecules in the microribbon cavity.

Supplementary Figure 13 | AR μ-PL spectrum of the PDI-O microribbon with width of 4.8 μm.

Supplementary Note 1 | Calculation of the polariton energies via the coupled oscillator model.

Supplementary Note 2 | Calculation of refractive index.

Supplementary Figure 14 | PL intensity as a function of pump fluence of PDI-O microcrystals.

Supplementary Figure 15 | A sketch of Young's double-slit interferometry double-slit experiment setup and the recorded intensity profiles of the microribbon.

Supplementary Figure 16 | Space-resolved AR μ-PL spectra of the PDI-O microribbon at 8.5 P_{th}.

Supplementary Figure 17 | PL images of PDI-O microribbon at different pump fluences.

Supplementary Note 3 | Discussion on repulsive interactions in polariton condensates.

Supplementary Disucussion

1. Photophysical properties of PDI molecules

The synthesis route of PDI-O is shown in **Supplementary Figure 1**. PDI-O contains a perylene diimide aromatic core and two 2,6-diisopropylphenyl groups substituted at imide position. The extensive π-conjugation and symmetric molecular structure leads to a large transition dipole moment (9.317 D) with direction along the N-N axis (**Supplementary Figure 2**). The ultraviolet-visible absorption (Abs) and photoluminescence (PL) spectra (**Supplementary Figure 3**) of dilute PDI-O solution show a narrow absorption linewidth (23 nm) and a small Stokes shift (10 nm), which is attributed to the planar and rigid molecular structure of PDI-O. The large oscillator strength, narrow absorption linewidth, and small Stokes shift are the basic requirements for strong coupling. In addition, the rigidity of the molecule suppresses internal conversion internal conversion caused by intramolecular motion, bringing about high quantum yield of fluorescence (90%), which is important for the realization of polariton Bose-Einstein condensation.

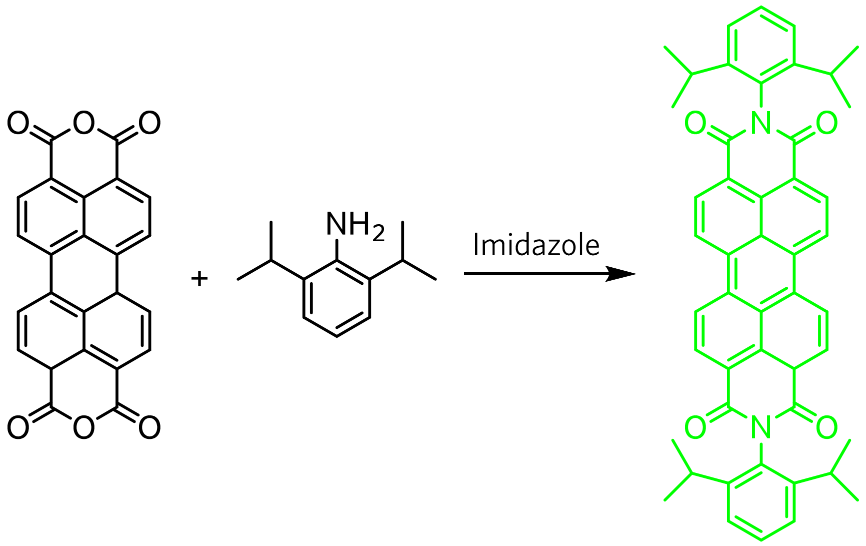

Supplementary Figure 1 | Synthesis route of PDI-O.

3,4,9,10-Perylenetetracarboxylic dianhydride (1.96 g, 5 mmol), 2,6-diisopropylaniline (2.07 mL, 11 mmol) and imidazole (10 g) were mixed and stirred at 140 °C for 4 h. The mixture was then diluted with ethanol, followed by the addition of HCl (2 M, 200 mL). The precipitate thus formed was collected through vacuum filtration and further purified by silica gel chromatography using dichloromethane as the eluent. The desired product (PDI-O) was obtained in 75% yield (2.70 g).

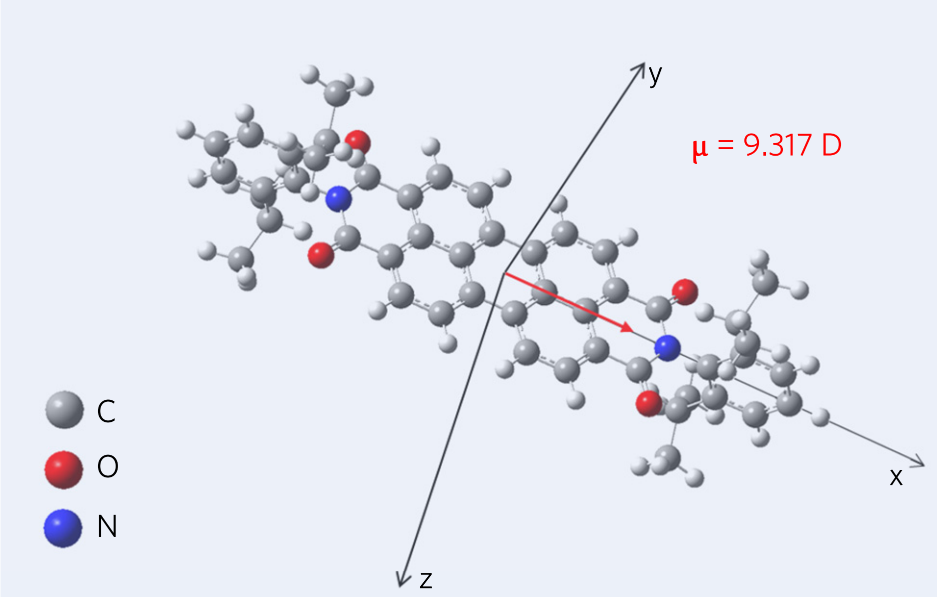

Supplementary Figure 2 | Calculated transition dipole moment vector (red arrow) of PDI-O from S_0 to S_1.

The components of transition dipole moment along the x, y and z axes are μ_x = 9.3172696 D, μ_y = 0.0000587 D and μ_z = 0.0000214 D, respectively. The calculated result reveals that PDI-O molecule has a transition dipole moment of 9.317 D, whose direction is along the N-N axis. The large transition dipole moment is favor for strong coupling because, according to the strong coupling theory, the coupling strength is proportional to the transition dipole moment[3].

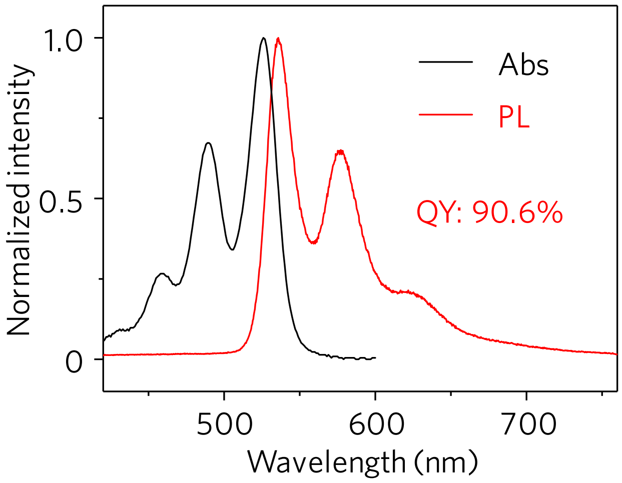

Supplementary Figure 3 | Normalized absorption and PL spectra of PDI-O in the dilute solution (dichloromethane).

The spectra show that the absorption maximum of PDI-O peaks at 525 nm with a narrow absorption linewidth of 23 nm, while its fluorescence maximum is redshifted to 535 nm. The narrow absorption linewidth and the small Stokes shift have been proven to be necessary for strong coupling, which suggests that PDI-O is an ideal compound for the generation of polaritons. Furthermore, the high quantum yield (QY) of fluorescence indicates that PDI-O can provide a large number of excitons available to form polaritons, which is important for the realization of polariton Bose-Einstein condensation.

2. Properties of the PDI-O microribbons

Morphology of PDI-O microribbons

The obtained microribbons exhibit uniform width (*W*) of several micrometers and thickness (*d*) in the range of one to a few hundred nanometers along the entire length (*L*) of several hundreds of micrometers (**Supplementary Figure 4**) The Atomic force microscopy (AFM) image (**Supplementary Figure 5**) of a PDI-O microribbon shows that the microribbon has smooth surfaces and flat side facets. The smooth surfaces would minimize the optical scattering loss, and the flat side facets can efficiently reflect guided light, which enables PDI-O microribbons to function as waveguide F-P microcavities along the ribbon width. The PL spectra of the PDI-O microribbon in transverse-electric (TE) polarization (with electric field perpendicular to the cavity direction) shows discrete set of resonant modes (**Supplementary Figure 6a**), indicating the cavity effect of the PDI-O microribbon. The Q factor of the cavity was estimated to be ranged from 130 to 170 for λ =585 nm using $Q=\lambda/\Delta\lambda$, where $\Delta\lambda$ is the full-width at half-maximum (FWHM) of the resonance peaks obtained from the fitted Lorentzian line shapes (**Supplementary Figure 6b**).

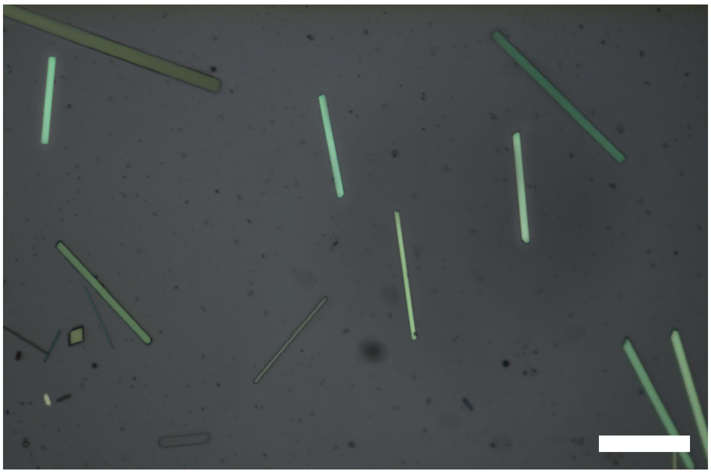

Supplementary Figure 4 | The bright-field image of a PDI-O microribbons. Scale bar: 60 μm.

The image shows that the as-prepared PDI-O microcrystals have a well-defined ribbon-like morphology with uniform widths of several micrometers along the entire length of several hundreds of micrometers. The uniform morphology and the clean surfaces of the PDI-O microribbons render them capable of forming natural microcavities.

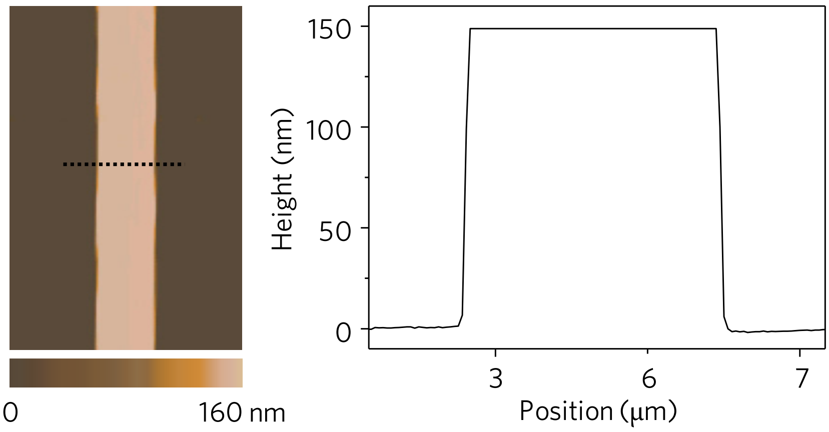

Supplementary Figure 5 | AFM image and the corresponding cross section profile of a single PDI-O microribbon. The scan area is 20 × 20 μm.

Supplementary Figure 5 shows that the PDI-O microribbon exhibits rectangular cross-section, defect-free surface, and flat side facets. These side facets can efficiently reflect guided light with minimized scattering loss, enabling the microribbons to function as lateral F-P cavities for strong coupling.

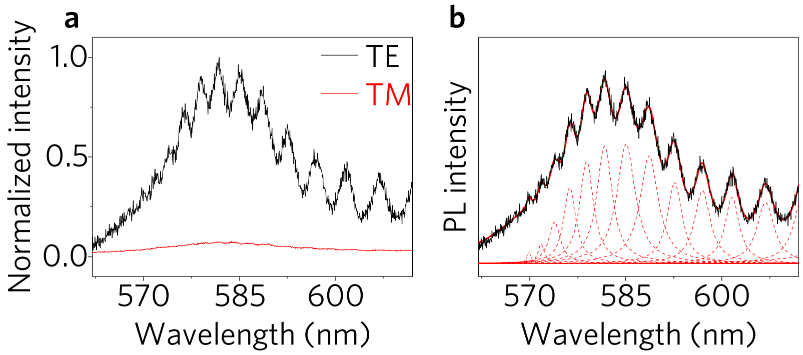

Supplementary Figure 6 | a, PL spectra of a PDI-O microribbon with TM and TE polarization. **b**, TE polarized PL spectrum of the PDI-O microribbon along with the fitted Lorentzian line shapes (dashed red lines) and overall fitting line (red solid line).

The microribbon shows negligible emission with transverse-magnetic (TM) polarization and strong TE-polarized emission, which suggests that the transition dipole moments are perpendicular to the ribbon width[2]. The clear multiple resonance peaks in TE-polarized spectrum confirm the cavity effect of the PDI-O microribbon which results from the long travelling of light in the material caused by total internal reflection. From Lorentzian fitting of the PL spectrum, we can obtain the full-width at half-maximum (FWHM) of the resonance peaks, which was used to calculate the Q factor of the cavity by $Q = \lambda/\Delta\lambda$[3].

Crystal structure of PDI-O microribbons

X-ray diffraction (XRD) patterns taken for the microribbons (**Supplementary Figure 7**) show sharp peaks, indicating that the microribbons are highly crystalline and have a monoclinic crystal structure. The monoclinic PDI crystal belongs to the space group of P 1 21/n1 with cell parameters of a = 10.027(2) Å, b = 19.069(4) Å, c = 1.494(3) Å, α = γ = 90°, β = 97.020(3)°. The PDI-O molecules adopt a stacking mode of J-type aggregation and maintain a "head-to-tail" orientation (**Supplementary Fig 8**), reducing non-radiative decay caused by π-π interactions and thus providing high-density Frenkel excitons[4]. The close proximity of adjacent PDI-O molecules gives rise to the formation of delocalized exciton states, whose transition dipole moment is constructed from a weighted vector sum of PDI-O transition dipole moments[5]. The plots of PL intensity as a function of excitation polarization angle (**Supplementary Figure 9**) shows that the PL intensity is strongest when the excitation polarization is parallel to the direction of the ribbon length, which reveals that the dipole orientation of excitons is perpendicular to the microribbon long axis. Such dipole orientation might maximize exciton-polariton coupling strength, because the light-matter coupling strength is proportional to the scalar product of transition dipole moment and the cavity electric field[2]. When excited with a laser spot, the PDI-O microribbon exhibits bright emission from both lateral facets (**Supplementary Figure 10a**), manifesting that the cavity modes in the ribbon width direction (defined as x-direction) are preferentially formed. The absence of cavity modes in the ribbon length (defined as y-direction) is attributed to (i) the directionality of the dipole

emission (**Supplementary Figure 10b**), (ii) the large optical loss induced by re-absorption of the PDI-O molecules during the long-distance propagation of light in the y-direction, and (iii) the weak light confinement resulted from the refractive index increase at the excitation area. To verify this conclusion, we have performed numerical simulation of the electric field intensity distributions in the microribbon. The microribbon with a background refractive index n_{bg} = 2.2 + 0.01i was excited with a dipole source located at the center, and the refractive index near the source was set to be slightly higher than n_{bg} (n = 2.3). Note that the imaginary part of n_{bg} indicates the absorption-induced optical loss. **Supplementary Figure 10c** shows the simulated electric field intensity distributions in the microribbon, from which we can find that light is confined in the y-direction and coupled into cavity modes in the x-direction.

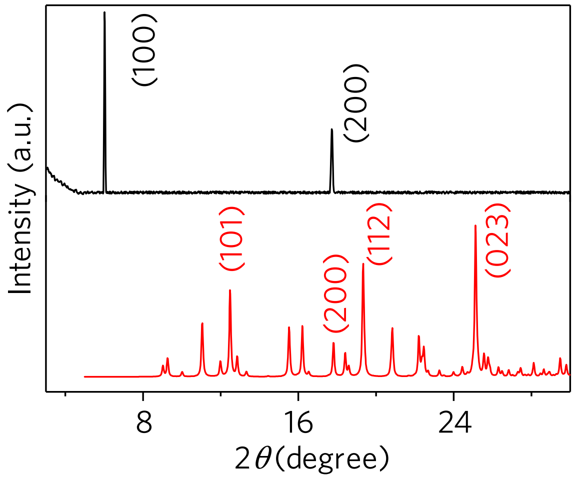

Supplementary Figure 7 | XRD patterns of the PDI-O microribbons (black) and stimulated PDI-O crystals (red).

The diffraction peaks in the XRD pattern of the PDI-O microribbons well match those of the simulated results from the single-crystal diffraction of PDI-O, which is indexed to a monoclinic single crystal (space group: P 1 21/n1, a = 10.027(2) Å, b = 19.069(4) Å, c = 1.494(3) Å, α = γ = 90°, β = 97.020(3)°). This reveals that the PDI-O microribbons are highly crystalline structures. The XRD pattern of the as-prepared microribbons which are all lying on the substrate (**Supplementary Figure 4**) exhibits only diffraction peaks corresponding to the (100) crystal planes, indicating that the direction of the ribbon thickness is perpendicular to the (100) crystal plane.

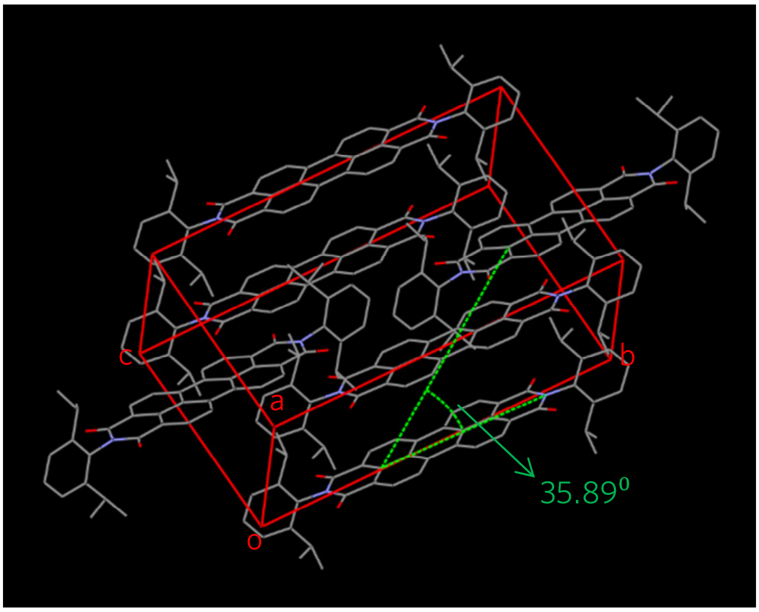

Supplementary Figure 8 | A unit cell of PDI-O crystal.

The PDI molecules in the crystal adopt a J-type stacking mode, as evidenced by the slipping angle between adjacent molecules θ =35.89°, which is less than the so-called "magic angle" θ_M = 54.7°. The J-type stacking mode would reduce non-radiative decay caused by π-π interactions, which is beneficial for providing sufficient number of excitons to form polaritons.

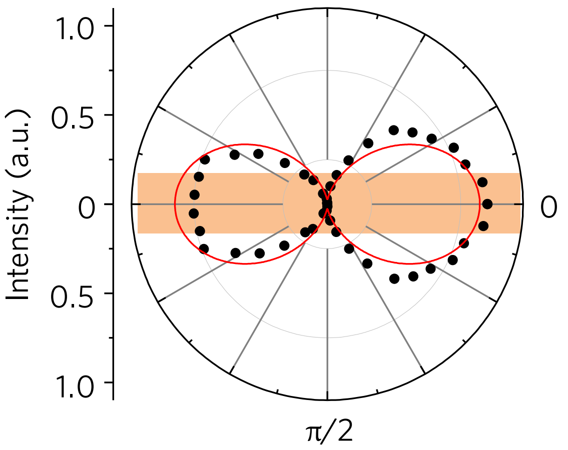

Supplementary Figure 9 | PL intensity as a function of excitation polarization angle with respect to the PDI-O microribbon long axis. The microribbon was excited by a 405 nm CW laser.

The plot was obtained by measuring PL intensity at different excitation polarization angle while keeping excitation power unchanged. As displayed in **Supplementary Figure 9**, the PL intensity is strongest at the polarization angle of zero, indicating that the exciton transition dipole moment is parallel to the microribbon long axis. This dipole orientation would maximize the coupling strength between excitons and photons, because the coupling strength is proportional to the scalar product of transition dipole moment and the cavity electric field[2].

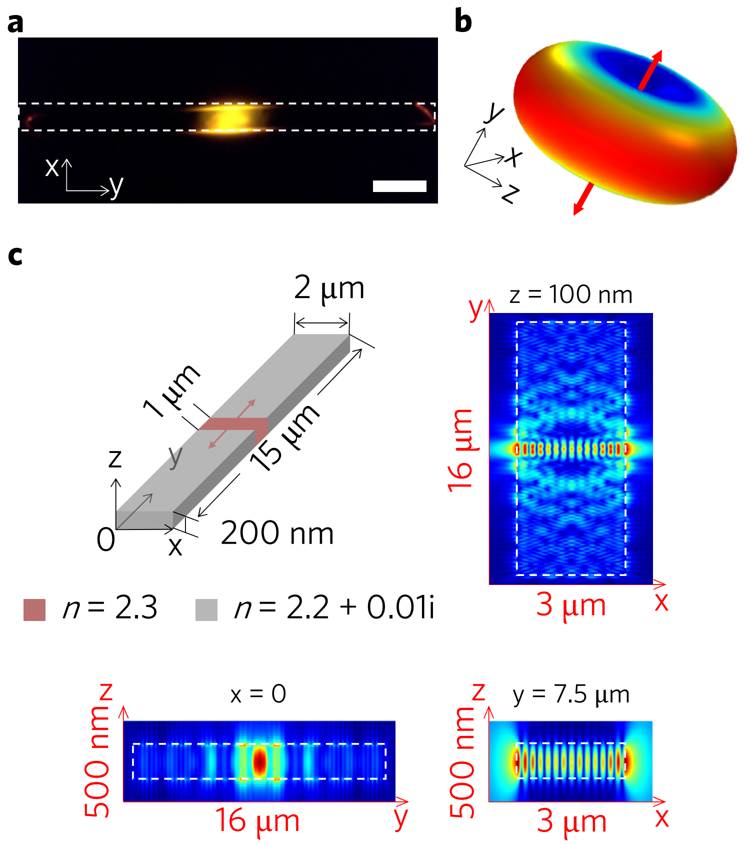

Supplementary Figure 10 | a, PL image of a microribbon excited by a laser spot. Scale bar: 10 μm. **b**, Stimulated emission profile of a dipole in the three-dimensional space. **c**, Simulated electric field intensity distribution in a microribbon with the dipole source (red double-headed arrow) located at the center (x =1 μm, y= 7.5 μm, z = 100 nm). The dipole is parallel to the y-direction. The refractive index near the dipole source is set to be n = 2.3, which is slightly larger than the background value (2.2) considering the thermal and excited-state refractive index changes under laser excitation. The refractive index outside the excitation area is taken as n = 2.2 + 0.01i for the introduction of loss due to re-absorption of PDI-O molecules. The white dashed squares in **a** and **c** indicate the profile of the microribbon.

The PDI-O microribbon excited with a laser spot exhibits bright emission from both lateral facets, indicating that the emission from PDI-O molecules (dipole sources) is coupled into the cavity modes in the width direction. The emission pattern of a dipole is torus-shaped and the emission intensity is strongest in the direction perpendicular to the dipole axis, which indicates that the coupling between the dipole and cavity modes is orientation dependent. Under the pump laser excitation, the energy deposited by the laser may lead to the variations of the material density and thus an increase of the refractive index at excitation area[6]. In addition, a large fraction of PDI-O molecules is pumped into the excited states, and the significant population redistribution would produce an additional increase in the refractive index[7]. The photoinduced refractive index increase further results in weak potential for light confinement in the y-direction. The simulated electric field intensity distributions (**Supplementary Figure 10c**) show that the emission of the dipole was confined in the y-direction and coupled into cavity modes in the x-direction, which agrees well with the experimental results (**Supplementary Figure 10a**). Therefore, the generated Frenkel excitons in the single-crystal PDI-O microribbons can efficiently undergo strong coupling with cavity photons, which facilitates the realization of polariton condensation.

3. Strong exciton-photon coupling in PDI-O microribbon cavities

The polariton emission is located near 580 nm, which indicates that the energetic separation between polariton ground state (**k**$_y$ = 0) and PDI-O molecular ground state (S_{00}) is 2.14 eV. This value equals to the energetic separation between exciton reservoir (S_{10}) and the first vibronic sublevel of the molecular ground state (S_{01}), indicating molecular vibration-assisted population of polaritons from the exciton reservoir, as shown in **Supplementary Figure 12**. The measured AR μ-PL spectrum was fitted by calculating the polariton energies (**Supplementary Note 1**) using the coupled oscillator model[8]. The fitting parameters were used to calculate the refractive index dispersion relation (**Supplementary Note 2**), which can also be calculated from experimental data (**Supplementary Note 3**). Both polariton energies and refractive index show good fit between experimental results and the coupled oscillator model, manifesting the system is in strong coupling regime. The background index of the waveguide cavity was obtained by measuring angle-resolved PL spectra of the PDI-O microribbon in the longer wavelength where measured dispersion corresponds to the uncoupled waveguide cavity modes as the photon energies are far away from exciton energy (**Supplementary Figure 13**). A background index of n_{bg} = 2.2 was obtained by fitting the dispersion to the equations discussed in **Supplementary Note 1**.

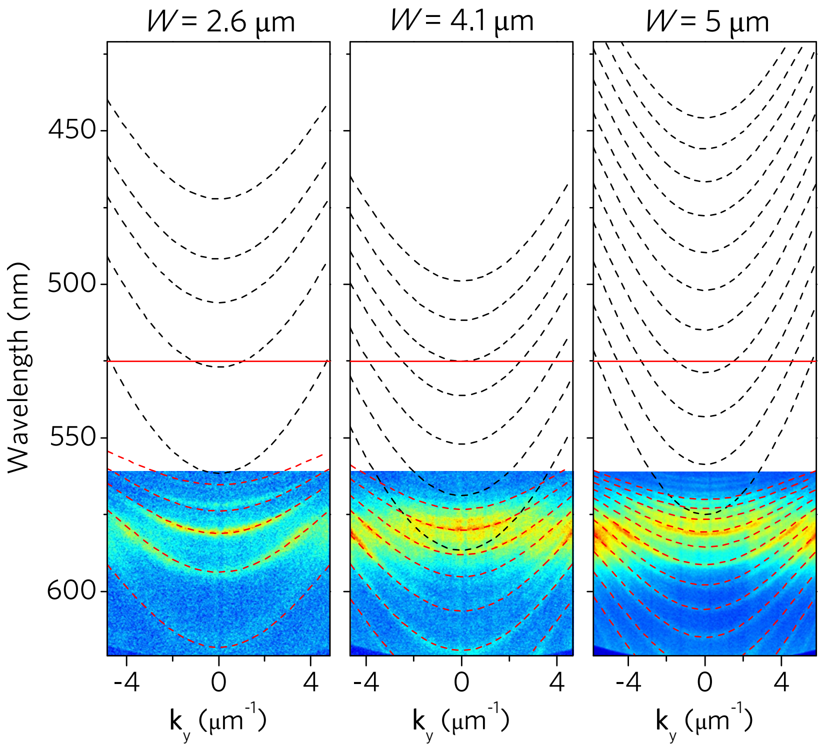

Supplementary Figure 11 | AR-μ-PL spectra of microribbons with different width (*W*) and thickness (*d*). The dispersions of polariton modes (red dashed lines) and bare cavity modes (black dashed lines) are obtained from fitting the measured dispersion to a coupled harmonic oscillator model. Red solid lines represent exciton energy.

The PL spectra exhibit clear multiple modes with unequal mode spacing and smaller curvatures at short wavelengths, indicating the occurrence of strong coupling between excitons and the multiple cavity modes. The polariton branches are dependent on the width and thickness of the PDI-O microribbons, which offers an approach to discrete tuning of the polariton modes and thus the condensate energies.

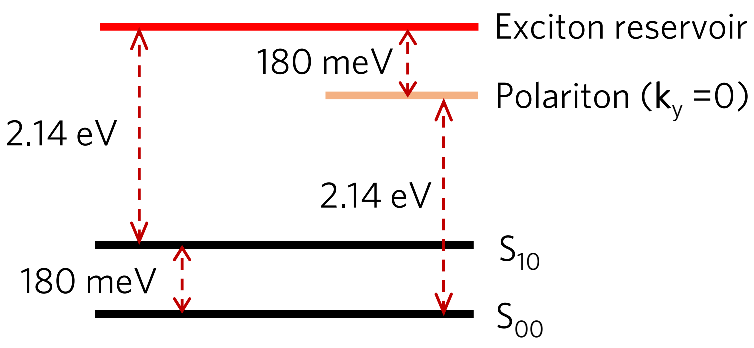

Supplementary Figure 12 | Relevant energy levels of PDI-O molecules in the microribbon cavity.

From the absorption and PL spectra of PDI-O molecules in **Supplementary Figure 3**, we can find that the absorption peaks are located at 2.36 eV (525 nm) and 2.53 eV (489 nm), corresponding to the $S_{00}\rightarrow S_{10}$ and $S_{00}\rightarrow S_{11}$ transitions respectively. Meanwhile, the emission spectrum is a mirror image of the absorption spectrum showing the main emission peak at 2.32 eV (535 nm) and a vibronic replica at 2.14 eV (580 nm), which correspond to the $S_{10}\rightarrow S_{00}$ and $S_{10}\rightarrow S_{01}$ transitions respectively. The vibronic energy was thus estimated to be 2.32 eV – 2.14 eV = 180 meV. As shown in **Supplementary Figure 12**, the energetic separation between the polariton ground state ($\mathbf{k}_y = 0$) and the PDI-O molecular ground state (S_{00}) is 2.14 eV (580 nm, from the polariton emission spectrum), and therefore the polariton ground state is energetically lower than the exciton reservoir by 180 meV, indicating that the polariton ground state is directly populated with emission of a vibron.

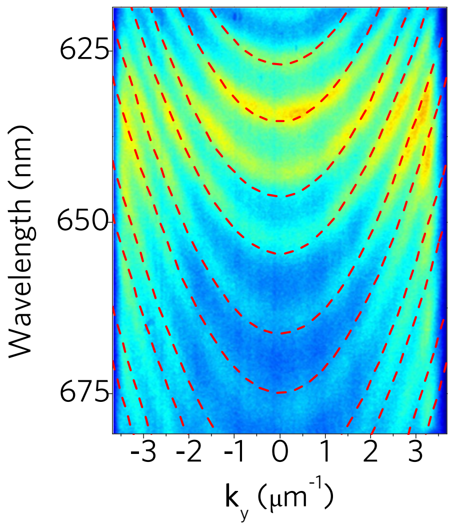

Supplementary Figure 13 | AR μ-PL spectrum of the PDI-O microribbon with width of 4.8 μm and thickness of 243 nm. Dashed curves are fitted dispersions of uncoupled waveguide cavity modes.

The PL wavelength is in the range from 620 to 680 nm, which is ~100 nm longer than the absorption maximum of PDI-O (525 nm). Exciton-photon strong coupling did not occur due to the large energy separation between them, and therefore the measured dispersion corresponds to the uncoupled waveguide cavity modes. By using the equations discussed in **Supplementary Note 1**, we can give a best fit to the measured results with fitting background index $n_{bg} = 2.2$.

Supplementary Note 1 | Calculation of the polariton energies via the coupled oscillator model.

The coupled oscillator model is expressed by[9]

$$\begin{pmatrix} E_c & \Omega/2 \\ \Omega/2 & E_{ex} \end{pmatrix}\begin{pmatrix} \alpha_c \\ \alpha_{ex} \end{pmatrix}=E_p\begin{pmatrix} \alpha_c \\ \alpha_{ex} \end{pmatrix} \quad (1)$$

Where E_c, E_{ex} and E_p are the uncoupled cavity mode energies, exciton energy, and polariton energies, respectively. Ω is Rabi splitting energies indicating the coupling strength. The mixing coefficients α_c^2 and α_{ex}^2 describe the relative photonic and excitonic weightings of the polaritons. In our PDI-O microribbon, the exciton energy extracted from the absorption spectrum is 2.36 eV.

The thickness of the microribbons is in the range of one to a few hundred nanometers, which is large enough for the microribbon to function as a slab waveguide and support guided modes. For the guided modes which travel inside the microribbon in x-direction by total internal reflection in the zigzag fashion, the sum of all phase shifts after each round trip of the wave must be equal to a multiple of 2π, which can be expressed by the characteristic equation:[9]:

$$2k_z d\text{-}2\varphi_t\text{-}2\varphi_b=2m\pi \quad (2)$$

Where k_z is the wave vector inside the microribbon along the z-axis, and φ_t and φ_b are phase shifts on total reflection from the microribbon's top and bottom facets, respectively. m and d are the mode order of the guided modes and thickness of the microribbon.

The z-component of the mode wave vector k_z can be expressed by:

$$k_z=\sqrt{n_{bg}{}^2k_0{}^2-\beta^2} \tag{3}$$

Where $k_0 = 2\pi/\lambda$ and n_{bg} are the wave vector in free space and the background refractive index of the microribbon, respectively. $n_{bg}k_0$ and β are the wave vector of the guided modes and the propagation constant along the x-axis, respectively. For a microribbon with a width of W, β satisfies the F-P resonance condition

$$\beta=\frac{N\pi}{W} \tag{4}$$

Where N is integer number for modes.

For TE modes, we can extract from the Fresnel formulas the following expressions for the phase shifts φ_t and φ_b:

$$\tan\varphi_t=\frac{\sqrt{\beta^2-n_{air}{}^2k_0{}^2}}{\sqrt{n_{bg}{}^2\mathrm{k}_0{}^2-\beta^2}} \tag{5}$$

$$\tan\varphi_b=\frac{\sqrt{\beta^2-n_{sub}{}^2k_0{}^2}}{\sqrt{n_{bg}{}^2k_0{}^2-\beta^2}} \tag{6}$$

Where n_{air} =1 and n_{sub} = 1.46 are the refractive index of the air and glass substrate, respectively.

For the waveguide cavity modes in the x-direction, considering the long length of the microribbon, the y-component of the wave vector k_y is free and can be expressed by[10]

$$k_y=k_0\tan(\arcsin\frac{\sin\theta}{n_{bg}}) \tag{7}$$

Therefore, the energy of the uncoupled F-P type waveguide cavity mode is

$$E_c = \frac{hc}{2\pi}\sqrt{{k_0}^2 + {k_y}^2} \tag{8}$$

Using these equations, we can give a best fit to the AR μ-PL mapping data for all cavity modes simultaneously with fitting parameters n_{bg} = 2.20 and Ω = 530 meV.

Supplementary Note 2 | Calculation of refractive index from fitting parameters in the coupled oscillator model.

Calculation of refractive index from experimental data

In F-P microcavities, the refractive index is calculated by[11]

$$n(\lambda)=\frac{\lambda^2}{2W\Delta\lambda} \tag{9}$$

Where λ is the wavelength of light, $\Delta\lambda$ is the space between the F-P mode peaks, and W= 5 μm is the width of PDI-O microribbon.

Calculation of refractive index from fitting parameters in the coupled oscillator model

The dielectric constant under strong coupling effect can be calculated by the following equations[10,11]

$$\Omega=\sqrt{2E_{\mathrm{ex}}(\omega_{\mathrm{L}}\text{-}\omega_{\mathrm{T}})} \tag{10}$$

$$\varepsilon(\omega)=\varepsilon_{\mathrm{bg}}\left(1+\frac{{\omega_{\mathrm{L}}}^2\text{-}{\omega_{\mathrm{T}}}^2}{{\omega_{\mathrm{T}}}^2\text{-}\omega^2\text{-i}\omega\gamma}\right) \tag{11}$$

Where ω_{L} and ω_{T} are the transverse and longitudinal resonance energies, respectively. $\varepsilon_{\mathrm{bg}} = {n_{\mathrm{bg}}}^2$ and γ are the background dielectric constant and damping constant. With Rabi splitting Ω (530 meV), exciton energy E_{ex} (2.36 eV) and background dielectric constant $\varepsilon_{\mathrm{bg}}$ (2.2^2) fixed, we fitted the refractive index. The corresponding parameters are ω_{L} = 2.264 eV (573.0 nm), ω_{T} = 2.15 eV (576.8 nm), and γ =7 meV. The large

transverse longitudinal splitting energy (ω_L - ω_T = 114 meV) indicates the large exciton oscillator strength of organic materials.

4. Bose-Einstein condensation of exciton-polaritons

The threshold for photon lasing was estimated by measuring the ASE of PDI-O microcrystals without cavity effects. **Supplementary Figure 14a** is the input-output curves of irregularly shaped PDI-O microcrystals exhibiting no cavity effect, from which we obtained the threshold for ASE of ~15 μJ cm^{-2}. We have measured input-output curves of the microribbons at high pump power to observe the threshold for photon lasing, but unfortunately no another threshold appears near the pump fluence of 15 μJ cm^{-2}, and the PL intensity starts to decrease with increasing pump power due to material degradation (**Supplementary Figure 14b**)., We believe that the absence of the second threshold is ascribed to the efficient exciton-polariton strong coupling which transforms excitons into stable polaritons. An important characteristic of BEC is the emergence of long-range spatial coherence which was probed in the microribbons by Young's double-slit interferometry experiment (**Supplementary Figure 15a**). The obvious interference fringes with the visibility contrast of 35% (**Supplementary Figure 15b**) indicate a long-range order of spatial coherence has been established in the microribbon.

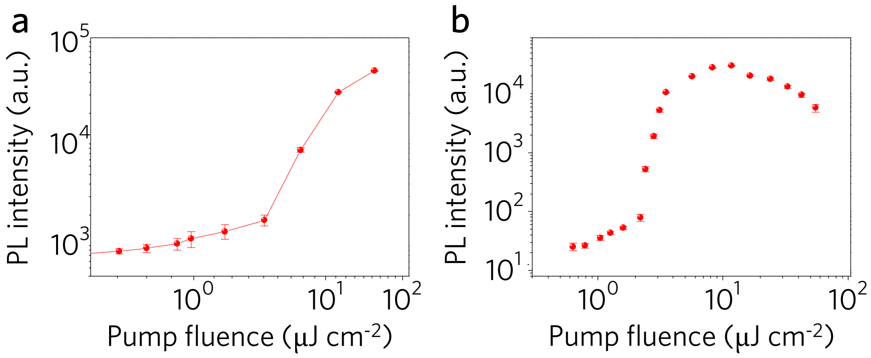

Supplementary Figure 14 | PL intensity as a function of pump fluence of a PDI-O microcrystal without cavity effect (**a**) and a regular PDI-O microribbon (**b**). Error bars indicate the 95% confidence intervals obtained from the fits to the measured PL spectra.

The threshold for photon lasing estimated by measuring the ASE of PDI-O microcrystals without cavity effects is ~15 μJ cm^{-2}. The input-output curves of the PDI-O microribbons shows that at such pump power the PL intensity starts to decrease, and no second threshold for photon lasing appears.

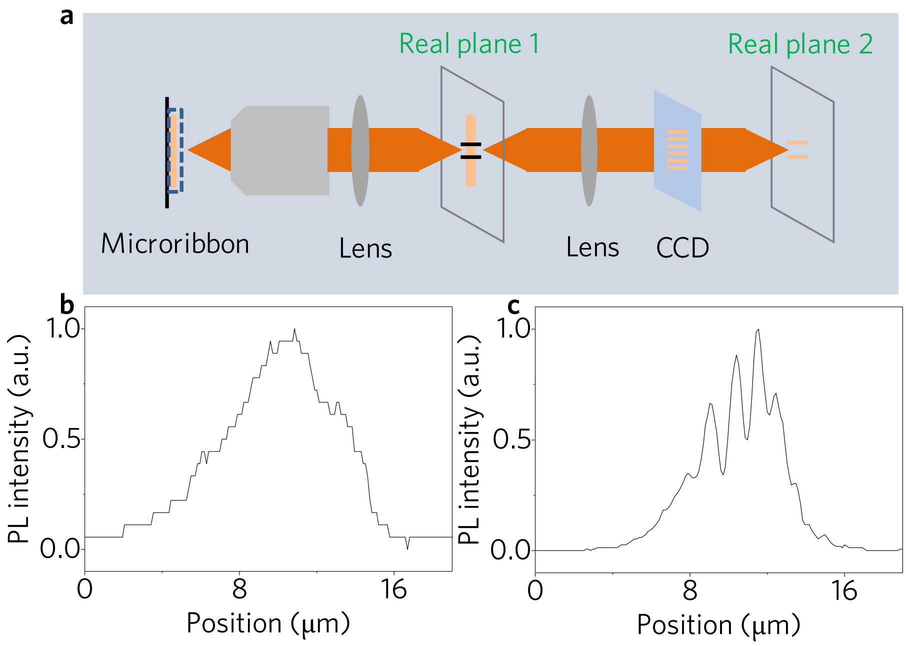

Supplementary Figure 15 | a, A sketch of Young's double-slit interferometry experiment setup. Intensity profile of the microribbon recorded below (**b**) and above (**c**) the threshold.

Supplementary Fig 15a is the schematic of Young's double-slit interferometry experiment, where the magnification coefficient at real plane 1 is 50, and the slit separation is 60 μm. The CCD records the interference pattern of the microribbon's emission passing through the double slit. Below threshold, the interference fringes are barely visible. In contrast, distinct interference fringes are clearly observed above threshold with the visibility contrast of 35%, indicating the emergence of spatial coherence of BEC in the microribbon.

5. Repulsive interactions in polariton condensates

The repulsive interactions have been demonstrated in inorganic semiconductor quantum well microcavities at cryogenic temperatures, due to the Mott transitions at higher temperature[12,13]. In our PDI-O microribbons the Frenkel excitons exhibit large binding energy and delocalized nature resulted from J-aggregation, and therefore the repulsive interactions exist at room temperature. From the PL images of PDI-O microribbon (**Supplementary Figure 16**) we can see that after the pump fluence was decreased from 8.5 P_{th} to 1.0 P_{th}, the microribbon shows uniform emission, which excludes photo-induced damage as a cause of disappearance of emission at the excitation area, further confirming that the emission is from the propagated polaritons. The space-resolved AR μ-PL spectra (**Supplementary Figure 17**) shows that the wave vectors are unidirectional with the direction pointing away from the excitation area, suggesting that it is caused by the repulsive force from the excitation area. The repulsive interactions between polaritons and the reservoir of excitons at high pump power are discussed in **Supplementary Note 3**.

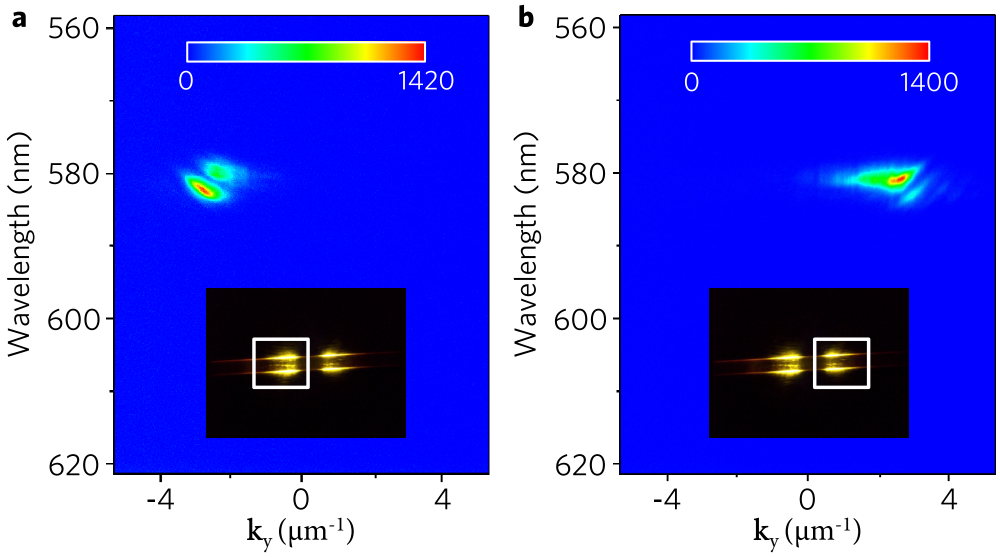

Supplementary Figure 16 | Spatial-resolved AR μ-PL spectra of different areas of the PDI-O microribbon measured at 8.5 P_{th}. The AR μ-PL spectra are collected from the selected areas marked in the Inset PL images.

From PL images and the space-resolved spectra, we can see that the emissions are unidirectional, and the emission directions deviate toward the side opposite to the excitation point. This indicates that the polaritons have acquired a wave vector in the direction away from the excitation point, which is ascribed to the repulsive force from excitons in the excitation area.

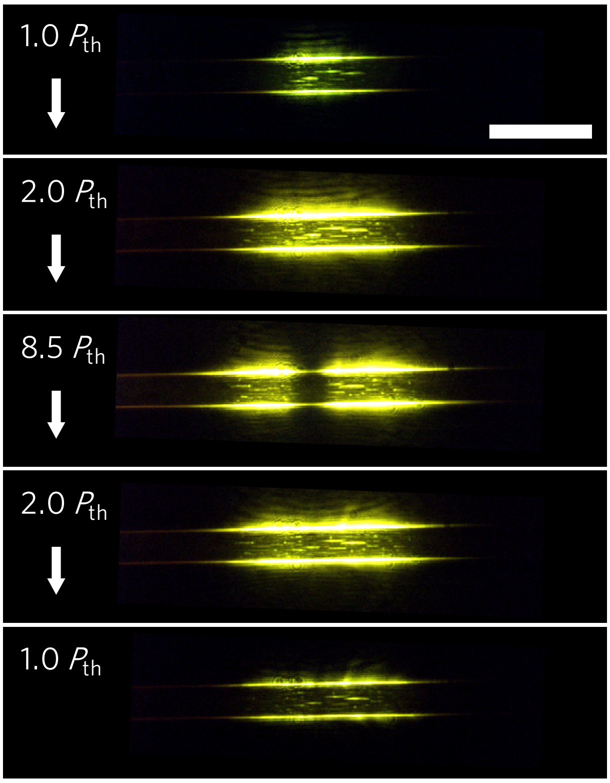

Supplementary Figure 17 | PL images of PDI-O microribbon at different pump fluences. Scale bar is 10 μm.

The PL images show that The uniform emission after pump power was decreased from 8.5 P_{th} to 1.0 P_{th}, the PDI-O microribbon still exhibits uniform emission in the excitation area. This result excludes photo-induced damage as the cause of disappearance of emission in the excitation area at 8.5 P_{th}, further confirming that the spatially separated emission results from the propagated polaritons driven by repulsive interactions in polariton condensates.

Supplementary Note 3 | Discussion on repulsive interactions in polariton condensates.

In steady-state polariton condensates, the macroscopic wave function $\psi(\mathrm{r})$ of polaritons can be described by the following mean-field Gross-Pitaevskii equation[13]:

$$\left\{-\frac{h^2}{2m}\nabla^2+g_{\mathrm{res}}n_{\mathrm{res}}(\mathrm{r})+g|\psi(\mathrm{r})|^2\right\}\psi(\mathrm{r})=\upsilon\psi(\mathrm{r}) \quad (12)$$

Where m is the effective mass of polaritons, and υ is chemical potential of polaritons. g and g_{res} describe the strength of polariton -polariton and polariton-exciton reservoir interactions, respectively. $n_{\mathrm{res}}(\mathrm{r})$ is exciton reservoir density controlled by the pump and reservoir decay rate. The spatial distribution of exciton reservoir $n_{\mathrm{res}}(\mathrm{r})$ is given by that of pumping due to the small diffusion coefficient of excitons. The inhomogeneous spatial distribution of exciton reservoir can induce spatial modifications of polariton wave function. Specifically, as there are no excitons outside the excitation area, the repulsive interactions between polaritons and excitons in the excitation area create a force, which expels polaritons from the excitation area. As a result, the polariton condensate undergoes a lateral acceleration and acquires a wave vector along the PDI-O microribbon[13].

Due to the small width and thickness of the microribbon, polaritons are confined in two dimensions, and therefore we can use a one-dimensional (1D) model to describe the polariton condensate with interaction potential. We have fitted the PL intensity

profiles according to a 1D Gross-Pitaevskii equation where the repulsion between polaritons and excitons $V_{res}(r)$ has a Gaussian profile and pump power-dependent intensity. The fitted polariton effective mass (m: ~2×10^{-35} kg) and repulsive interaction potential ($V_{res}(r)$: in order of 0.001 eV) are consistent to the previous report on organic polariton condensates[14]

.

Supplementary References

1. Hertzog, M., Wang, M., Mony, J. & Borjesson, K. Strong light-matter interactions: A new direction within chemistry. *Chem. Soc. Rev.* **48,** 937-961 (2019).
2. Bao, Q. *et al.* Polarized emission and optical waveguide in crystalline perylene diimide microwires. *Adv. Mater.* **22**, 3661-3666 (2010).
3. Dong, H. *et al.* Broadband tunable microlasers based on controlled intramolecular charge-transfer process in organic supramolecular microcrystals. *J. Am. Chem. Soc.* **138**, 1118-1121 (2016).
4. Wurthner, F., Kaiser, T. E. & Saha-Moller, C. R. J-aggregates: From serendipitous discovery to supramolecular engineering of functional dye materials. *Angew. Chem. Int. Ed.* **50**, 3376-3410 (2011).
5. Kasha, M., Rawls, H. R. & Ashraf El-Bayoumi, M. The exciton model in molecular spectroscopy. *Pure Appl. Chem.* **11**, 371-392 (1965).
6. Baum, A. *et al.* Pulse-duration dependency of femtosecond laser refractive index modification in poly(methyl methacrylate). *Opt. Lett.* **33**, 651-653 (2008).
7. Price, M. B. *et al.* Hot-carrier cooling and photoinduced refractive index changes in organic-inorganic lead halide perovskites. *Nat. Commun.* **6**, 8420 (2015).
8. Lidzey D.G., Coles D.M. Strong Coupling in Organic and Hybrid-Semiconductor Microcavity Structures. In: Organic and Hybrid Photonic Crystals. (Springer, Cham, 2015).

9. Kogelnik H. Theory of Dielectric Waveguides. In: Integrated Optics. Topics in Applied Physics (Springer, Berlin, 1975).

10. Sun, L., Ren, M. L., Liu, W. & Agarwal, R. Resolving parity and order of Fabry-Pérot modes in semiconductor nanostructure waveguides and lasers: Young's interference experiment revisited. *Nano Lett.* **14**, 6564-6571 (2014).

11. Zhang, C. *et al.* Two-photon pumped lasing in single-crystal organic nanowire exciton polariton resonators. *J. Am. Chem. Soc.* **133**, 7276-7279 (2011).

12. Wertz, E. *et al.* Spontaneous formation and optical manipulation of extended polariton condensates. *Nat. Phys.* **6**, 860-864 (2010).

13. Ferrier, L. *et al.* Interactions in confined polariton condensates. *Phys. Rev. Lett.* **106**, 126401 (2011).

14. Daskalakis, K. S., Maier, S. A., Murray, R. & Kena-Cohen, S. Nonlinear interactions in an organic polariton condensate. *Nat. Mater.* **13**, 271-278 (2014).
